# Vascularity of primary and metastatic renal cell carcinoma specimens

**DOI:** 10.1186/1479-5876-11-15

**Published:** 2013-01-14

**Authors:** Saadia A Aziz, Joshua Sznol, Adebowale Adeniran, John W Colberg, Robert L Camp, Harriet M Kluger

**Affiliations:** 1 Department of the School of Medicine, Yale University School of Medicine, New Haven, CT, USA; 2 Department of Pathology, Yale University School of Medicine, New Haven, CT, USA; 3 Department of Urology, Yale University School of Medicine, New Haven, CT, USA; 4 Section of Medical Oncology, Yale Cancer Center, 333 Cedar St. WWW213, New Haven, CT, 06520, USA

**Keywords:** Renal cell carcinoma, Microvessel area, Angiogenesis

## Abstract

**Purpose:**

Anti-angiogenic therapies are among the most commonly used drugs in renal cell carcinoma. Tumor vascularity, defined by microvessel area, may be associated with response to these drugs. Clinical studies suggest that metastatic sites are more responsive than primary tumors. Our purpose was to characterize microvessel area (MVA) in matched primary and metastatic samples and in samples of different histologies.

**Methods:**

We employed a method of automated, quantitative analysis of *in situ* tumor components to identify the area of CD-34 staining endothelial cells within renal cell carcinoma tumors. MVA was assessed in corresponding primary and metastatic samples from 34 patients, as well as in 334 primary nephrectomy specimens with variable histologies.

**Results:**

MVA measurements from different parts of the same tumor correlated well (R = 0.75), indicating that MVA was fairly uniform within a tumor. While MVA was slightly higher in primary tumors than corresponding metastatic sites, the difference was not statistically significant (P = 0.1). MVA in paired primary and metastatic samples correlated moderately well (R = 0.36). MVA was higher in clear cell than papillary histology and oncocytomas (P < 0.0001 and P = 0.018, respectively).

**Conclusions:**

Lack of significant differences MVA in matched primary and metastatic samples suggests that both types of tumors should respond to anti-angiogenic drugs. This should be confirmed on additional cohorts. Given the small cohort, future predictive biomarker studies entailing MVA measurements should include specimens from both sites. Clear cell carcinomas are more vascular than other histologic subtypes, which may explain the higher response rates to anti-angiogenic therapies in clear cell tumors.

## Background

Renal cell carcinoma (RCC) is fairly common, with an estimated incidence of 64,000 in the United States in 2012 [1]. Clear cell RCC (ccRCC) is the most prevalent subtype, reflecting roughly 80% of RCC tumors [2]. RCC tumors tend to be highly vascular [3]. Studies of tumor neovasculature have revealed silencing of the tumor suppressor von Hippel-Lindau (VHL) gene or loss of chromosome 3p, causing activation of hypoxia-inducible transcription factor, and further production of proangiogenic growth factors, such as vascular endothelial growth factor (VEGF) [4,5]. Angiogenesis is critical for sustaining neoplastic growth and hematogenous dissemination [6,7]. In the past decade, anti-angiogenic therapies have been shown to be beneficial in the treatment of advanced metastatic RCC, including the VEGF targeting drug, bevacizumab, given in conjunction with interferon, and the VEGF-R2 targeting drugs sorafenib, sunitinib, pazopanib and axitinib [8-12]. At present, no predictive biomarkers are available for selection of patients for these drugs. Seeing that they target angiogenesis, tumor vascularity may be associated with response to therapy. Our purpose was to determine patterns of tumor vascularity in historical samples and to compare vessel density in primary and metastatic RCC tumors.

Response of primary tumors to angiogenesis targeting agents is variable, however highly sensitive cases (complete responses) are relatively uncommon. Several groups have reported significant primary tumor debulking with pre-nephrectomy anti-angiogenic therapy in metastatic RCC patients [13-16]. However, a recent retrospective review showed less decrease in primary tumor diameter in metastatic RCC patients than in metastatic sites [17]. It is unclear whether there are differences in vessel density in primary and metastatic RCC tumors, and whether this may be the cause of possible discordant response in primary and metastatic sites.

The association between tumor vascularity and response to VEGF and VEGF receptor targeting drugs remains unclear. In a small pilot study, vascular permeability measured radiographically was significantly lower after sorafenib treatment, and this correlated well with time to progression (P = 0.01). Elevated baseline tumor vascular permeability correlated with improved progression free survival (P = 0.003), but not with radiographic decrease in tumor size. This study included 17 patients and definitive conclusions cannot be drawn [18]. A similar situation has been seen with treatment with sunitinib, where dramatic decreases in vascularity have been seen with little change in tumor size, and new response criteria based on vascular permeability are being studied [19].

Limited prior publications have evaluated tumor vascularity in RCC specimens and the association with VHL mutational status and prognosis. VHL mutation, particularly loss of function mutation, has been shown to be an independent prognostic factor in ccRCC. Contradictory results have been published on the role of microvessel density (MVD) and VHL mutational status. One small study of 40 cases showed higher levels of MVD in tumors with VHL mutations, while other studies show no significant correlation between mutational status and MVD [20-23]. Rioux-Leclercq et al. used standard immunohistochemical staining for tumor vessels and showed that high tumor vessel density is associated with poor outcome, while Imao et al. used similar methods on a small cohort of specimens and showed the inverse association [24,25]. Conversely, MacLennan et al. found that while there was no association between microvessel density and prognosis in ccRCC, microvessel densities were higher in clear cell and chromophobe histologies [6]. Two additional groups characterized associations between vessel density and pathological features and found an association between high microvessel area (MVA) and high stage and grade [26,27]. Microvessel area is defined by an automated quantitative method as the total area of microvessel in a given sample area. Microvessel density is defined as countable vessels in a sample area, as defined by Mlynek et. al [28]. A study by Sullivan et. al in breast cancer showed high correlation between MVA and MVD [29]. Yildiz et al. reported an inverse relationship between microvessel density and microvessel invasion and metastasis [30]. A major limitation of all of these studies is use of non-quantitative immunohistochemistry and small patient cohorts of less than 70 cases, contributing to conflicting results. Mertz et al. therefore conducted a more comprehensive study employing an automated, quantitative method to assess vessel density applied to a large cohort of 284 clear cell RCC tumors, and found that MVA was associated with improved survival [3]. Our group subsequently validated this finding in a cohort of over 300 nephrectomy specimens using the same automated method, and found that high MVA was associated with improved 10 year disease specific survival (HR = 0.87, P = 0.04) [31]. Okon et al. studied MVA in over 100 RCC primary tumors in a quantitative fashion and found that MVA was higher in ccRCC [32].To date no studies have assessed the differences in MVA in corresponding primary and metastatic specimens or in different histologic RCC subtypes in a quantitative fashion.

Given the potential association between the degree of tumor vascularity and response to VEGF or VEGR receptor targeting therapy, our primary purpose was to determine whether differences exist in MVA in matched primary and metastatic sites, particularly given that many patients have available archival specimens from either primary or the metastatic tumors, but not both. Furthermore, seeing that anti-angiogenic drugs are now used in non-clear cell RCC patients, we sought to determine whether there are differences in vessel density between the different histological subtypes of RCC, which may be a predictor for response. To analyze the microvessel area of tumor samples, we utilized our system of automated quantitative analysis (AQUA) which allows us to obtain more accurate objective measures of compartment (vessel) area within tissues [3,31,33]. Because of its ability to objectively assess biomarkers on a continuous scale, AQUA has been shown to outperform traditional "brown-stain" immunochemistry in several studies [33-35].

## Patients and methods

### Patient cohorts and tissue microarray (TMA) construction

Two non-overlapping cohorts were used for these studies: A cohort of matched primary and metastatic RCC cases and a cohort of sequential nephrectomy cases with stage I-IV disease. All tumor tissues were collected from the Yale University Department of Pathology Archives. Specimens and clinical information were collected with the approval of a Yale University institutional review board. Performance status, LDH, hemoglobin and calcium levels were not available. TMAs were constructed using standard methods with cores measuring 0.6 mm each.

#### Matched primary and metastatic RCC TMA

Thirty-four patients who had undergone both nephrectomy and metastatectomy between 1978 and 2011 were identified Histological subtypes included clear cell (91.2%) and mixed histology (two cases, 8.8%). One mixed histology case was a mixture of clear cell RCC with sarcomatoid changes and type 2 papillary RCC. The other was a mixture of type 2 papillary RCC and unclassified RCC with oncocytic, mucinous, and spindle cell features. Three percent were of Fuhrman grade I and IV, 38% grade II and 56% grade III. Age at diagnosis was 17–72 years (median-57). The time between nephrectomy and metastatatectomy ranged from 6 to 156 months, median 24 ± 39.5 months. Only two patients were treated with VEGF or VEGF-R targeting therapies. Metastatic sites included lung (13), bone (7), soft tissue, skin and lymph node (8), adrenal glands (2), liver (2), colon (1), and pituitary gland (1). Each tumor site was represented by four cores from different areas of the specimen; two cores from each tumor site were included in each of two TMA blocks.

#### Large cohort RCC nephrectomy TMA

Specimens were collected from 334 non-overlapping RCC patients who underwent nephrectomy between 1987 and 1999. This cohort has been described previously[31,36]. Histological subtypes included clear cell (74%), papillary (14%), mixed histology (4%), chromophobe (2%), and oncocytomas (6%). The mixed histology subset included clear cell with oncocytic features (2%) or with papillary features (2%). Age at diagnosis was 25–87 years (median-63). Among these patients, 56% had stage I disease, 8% had stage II and III each, and 28% had stage IV. Twelve percent were Fuhrman grade I, 52% grade II, 27% grade III and 9% grade IV. Tumors were represented by two cores placed in two TMA blocks.

### Immunofluorescent staining

Each slide was stained individually for CD-34, as previously described with a mouse monoclonal anti-human CD-34 antibody incubated overnight at a dilution of 1:100 [3,31]. CD-34 was used as a vessel (endothelial cell) marker based on studies by Yilmazer et al. which showed CD-34 immunohistochemical staining to be more specific and sensitive than CD-31 in determining microvessel density [37].

### Automated image acquisition and analysis (AQUA)

Images were acquired and analyzed using algorithms that have been previously described [33]. Monochromatic, high-resolution (1024 × 1024 pixel, 0.5 μm) images were obtained of each histospot using the 10X objective of an Olympus BX-51 epifluorescence microscope with an automated microscope stage and digital image acquisition driven by custom program and macro-based interfaces with IPLabs sofware (Scanalytics). Coalescence of Cytokeratin/CA-9/Streptavidin was used to localize the tumor compartment. Endothelial cells were distinguished from tumor cells by CD-34 expression. The percentage of CD-34 area within the tumor area was used to determine the MVA. Histospots were excluded if the tumor mask represented <3% of the histospot area or if there was anomalous staining (lacking DAPI or necrotic tissue).

### Statistical analysis

Statview and JMP 5.0 software were used (SAS Institute, Cary, NC). MVAs for replicate tumor cores were averaged. Associations between continuous MVA values and pathological parameters were assessed using ANOVA. Correlations between redundant histospots were assessed by Pearson linear regression.

## Results

### Measurement of microvessel area (MVA) by quantitative immunofluorescence analysis in RCC

**Figure 1:**
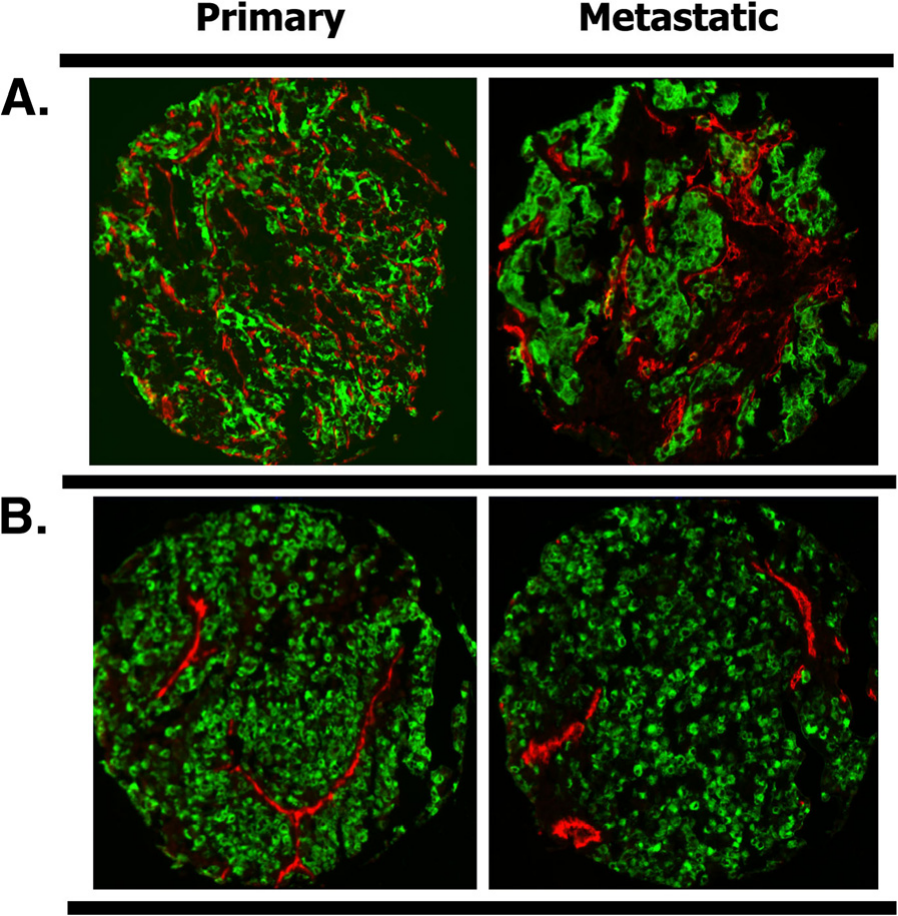
**High (panel A) and low (panel B) microvessel area (MVA) in matched primary and metastatic specimens by AQUA.** We used a cocktail of anti-cytokeratin and anti-carbonic anhydrase-9 conjugated to Cy2 to create a tumor mask (green), and anti-CD-34 conjugated to Cy5 (red) to identify microvessels. An example of a patient with high MVAs in both the primary and metastatic specimens is shown in panel **A**, and an example of low MVAs in both primary and metastatic specimens is shown in panel **B**. The corresponding MVA scores were 14.92% and 18.93%, respectively for panel **A** and 4.26% and 6.78%, respectively for the patient represented in panel **B**.

Given the role of angiogenesis in RCC, the area of CD-34 expressing cells within the tumor mask was measured in both the primary and metastatic tumors of 34 patients. Examples of high and low MVA in corresponding primary and metastatic specimens are shown in Figure 1. MVA distribution ranged from 0.44% to 25.19%, with a median MVA of 4.95% in these specimens.

### MVA in different areas of a given tumor

**Figure 2:**
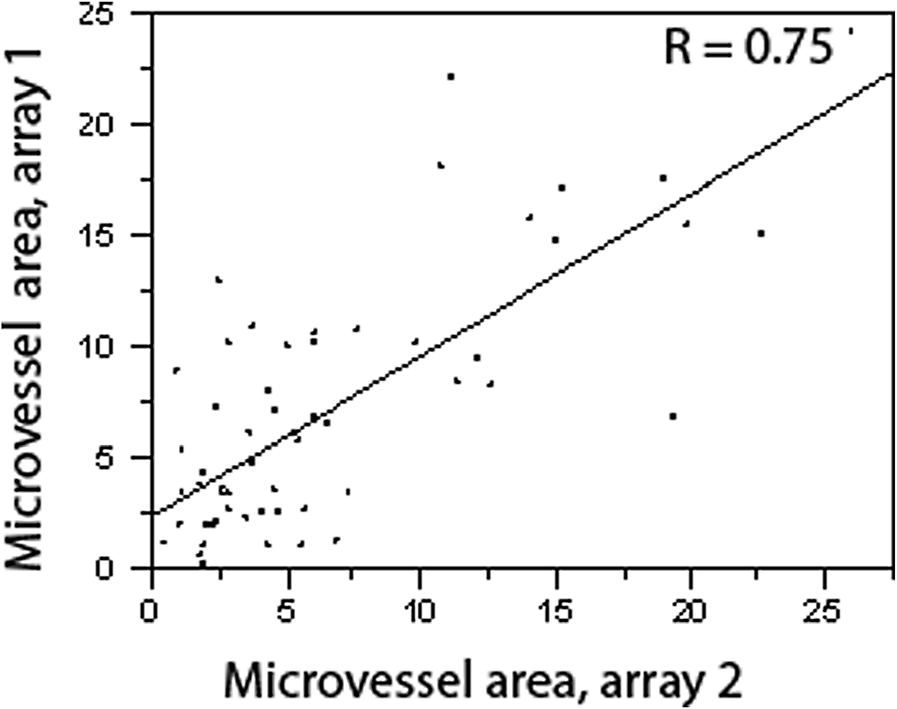
**Correlations between microvessel area (MVA) in different areas of a given tumor.** Two tissue microarrays, each containing duplicate histoscores taken from separate areas within the matched primary and metastatic RCCs, were constructed. Vessel Area, expressed as the percent area covered by CD-34 staining within the tumor, demonstrates good array-to-array correlation, and low intra-tumor variability, R = 0.75.

To assess intra-tumor heterogeneity in vessel density, we used four cores from the primary tumor and four cores from the metastatic tumors, placed on two separate sets of slides, each containing two cores from each site. MVAs from corresponding cores of each array were averaged to obtain a single concatenated value. The correlation between the values from each array was calculated using the Pearson test. Although some variability was seen, we found that the averaged values from the two arrays were highly correlated, R = 0.75 (P < 0.0001), as shown in Figure 2**,** indicating that the intra-tumor consistency in MVA is high.

### Comparison between MVA in matched primary and metastatic specimens

**Figure 3:**
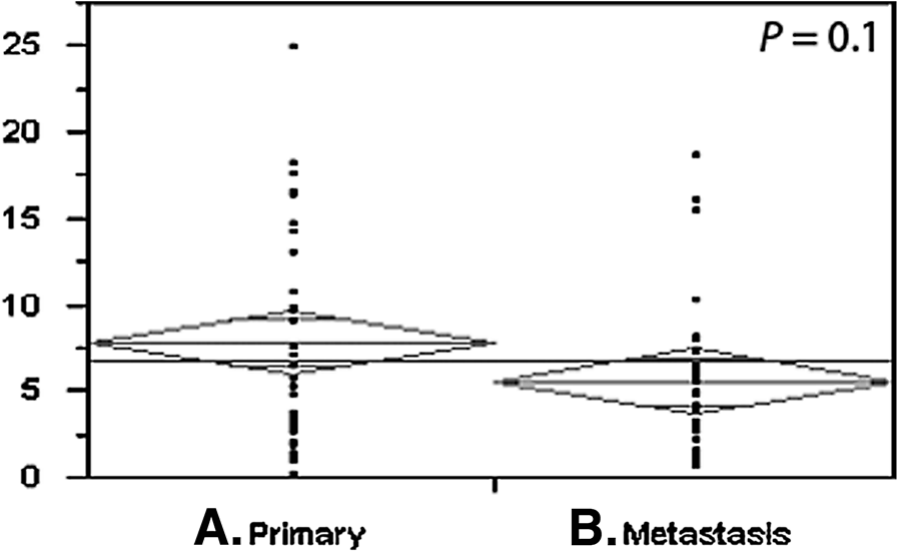
**Comparison between MVA in matching primary and metastatic samples.** Using paired t-tests, we compared MVA in 34 matched primary and metastatic specimens. As shown in the means plot, while the primary specimens were slightly more vascular, the difference was not statistically significant (P = 0.1).

**Figure 4:**
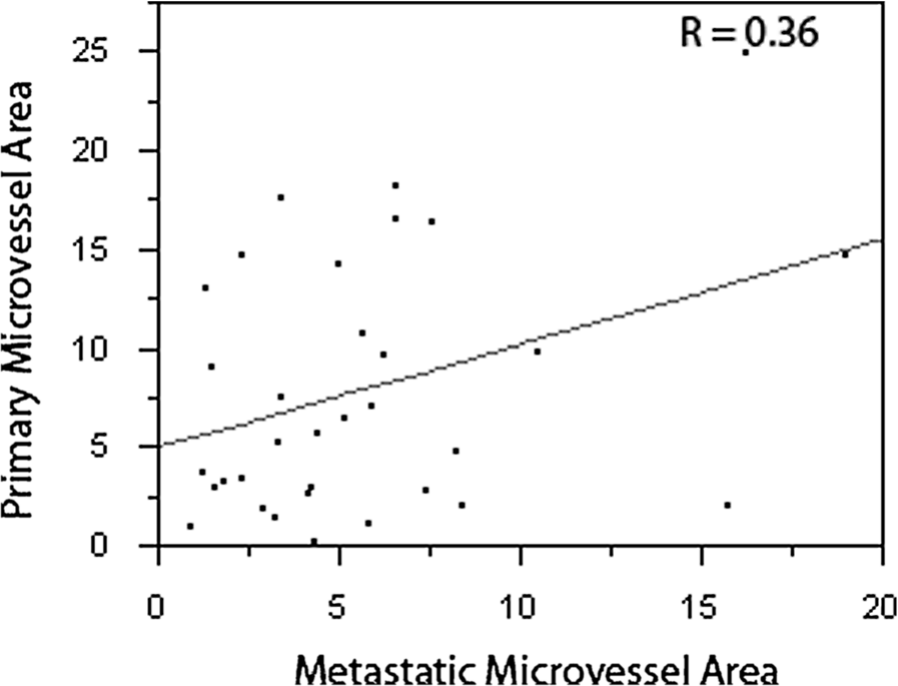
**Correlation between MVA in matched primary and metastatic samples.** Using the Pearson correlation test, we assessed the association between MVA in the two tumor types; while in some cases the association in strong, a fair degree of discordance was seen.

Using analysis of variance (ANOVA), we found that although the MVAs were minimally higher the primary specimens than their metastatic counterparts, there was no statistically significant difference (P = 0.1), as shown in the means plot in Figure 3. The mean MVA was 7.86% in primary tumors and 5.62% in metastatic tumors. To determine whether MVA in primary specimens can be used as a proxy to determine MVA in metastatic samples and vice-versa, we studied the correlation between MVA in matched primary and metastatic specimens using the Pearson correlation test. As shown in Figure 4, there is a moderate linear association between MVA in the two specimen types (R = 0.36), a number of cases had discordance between the primary and metastatic specimens.

### MVA in the different histologic subtypes

**Figure 5:**
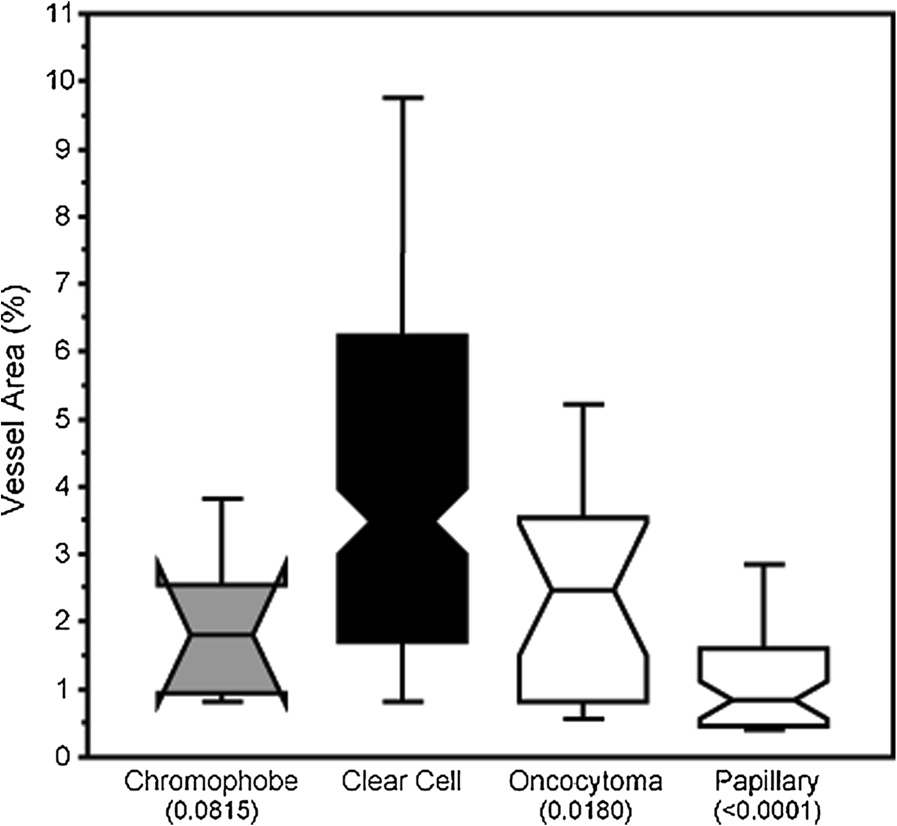
**Box plot demonstrating highest MVA in clear cell histological subtype.** MVA was studied in 334 primary RCC specimens of different histologic subtypes. While the MVA was variable in each subtype, clear cell carcinoma specimens had higher MVAs than papillary histology and oncocytomas.

Given that we only had 34 matched primary and metastatic tumors, we employed a larger historical cohort of primary nephrectomy RCC specimens to assess difference in MVA. This cohort includes clear cell, papillary, chromophobe, oncocytoma, and mixed histologies. MVA score distribution ranged from 0.1% to 25% (with a median value of 4.4%). The mean MVAs for the different subtypes were: 4.4% for clear cell, 1.28% for papillary, 1.98% for chromophobe, 0.99% for mixed histology, and 2.5% for oncocytomas. By ANOVA, we found that the clear cell subtype had significantly higher MVA than papillary histology and oncocytomas (P < 0.0001, and P = 0.018, respectively), as shown in Figure 5. Individual p-values were generated using the post-hoc Fisher PLSD (protected least significant difference) test assessed using an alpha of 5%. No significant differences were found between the other subtypes.

## Discussion

In this work we studied MVA in two patient cohorts; one cohort of matched primary and metastatic RCC specimens and a larger cohort of over 300 primary nephrectomy specimens. We found that MVA, when measured in a quantitative objective fashion, does not differ significantly in different areas of the tumor. Paired comparisons between the matched primary and metastatic sites revealed that the primary specimens are slightly more vascular, but the difference was not statistically significant. To determine whether MVA in a primary specimen accurately reflects that of corresponding metastases, we studied the correlation between MVA in the two tumor types and found that while there clearly is an association (R = 0.36), a fair degree of discordance was seen. We note that the range of time frames between nephrectomy and metastastatectomy was wide, and sample size of matched primary and metastatic specimens does not allow analysis of an association between MVA and time to metastatic disease. Moreover, our metastatectomy cohort might reflect patients with oligometastases amenable to local therapy, rather than widespread metastatic disease. Finally, using our larger cohort of primary nephrectomy specimens, we found that the clear cell carcinomas were significantly more vascular than papillary histology. In our previous work we showed that MVA is inversely correlated with Furhman grade, but not with stage. Similarly, it was associated with improved 10-year disease-free survival [31].

Although the first VEGF-R2 targeting drugs were approved for metastatic RCC over seven years ago, no predictive assays have been validated for any of these drugs to facilitate patient selection [8,9]. Vascular tumors should respond to anti-angiogenic therapy, and the discordance in responses of the primary and metastatic tissues is not well explained by MVD. Recent data published by Zhao et al. suggests that high MVD predicts better response to bevacizumab in non small cell lung cancer [38]. Attempts to identify predictors of response based on imaging studies in small groups of patients do suggest an association between increased baseline tumor vascularity by DCE-MRI and improved progression free survival (PFS) with sorafenib [18]. Ueno et al. used PET/CT on 30 patients treated with sunitinib or sorafenib and showed that baseline SUV uptake correlated with short progression PFS, while decreased SUV uptake after one month on therapy was a stronger predictor of PFS [39]. Clinical factors (standard laboratory values, performance status and time from diagnosis to treatment) do appear to be associated with improved PFS in patients treated with these drugs [40]. Models incorporating both clinical and radiographic criteria suggest that the combined model is superior to either modality alone [41]. Whether or not these factors are predictive of benefit from therapy as opposed to improved natural history of disease remains to be determined.

While the abovementioned studies focused on radiographic and clinical criteria, other early studies have attempted to determine the association between pre-treatment tumor-based characteristics and response to VEGF or VEGF-R targeting drugs. For example, two small retrospective cohort studies demonstrated an association between CAIX levels measured by immunohistochemistry and response to VEGF-R2 targeting drugs[42,43]. The purpose of the current study was to pave the way for future studies of associations between MVA and response to VEGF pathway targeted therapy. In previous studies we showed an inverse correlation between MVA and VEGF-R1 and –R2, but no significant correlation was found between MVA and VEGF [31]. Seeing that no clear association has been demonstrated between VEGF-R2 expression and response to VEGF-R targeted therapy, incorporation of MVA in biomarker studies may improve our ability to predict response. The majority of patients in the present study were not treated with these drugs, and the study was designed to determine baseline MVA characteristics in primary and metastatic RCC tumors. Seeing that most RCC patients in our institution have larger archival specimens from either the nephrectomy or the metastatectomy but not both (as evident by the small size of the matched primary and metastasis cohort relative to the nephrectomy cohort), our finding of differences in some (but not all) patients in MVA between primary and metastatic sites suggests that biomarker studies assessing MVA as a predictor of response should assess specimens from both sites. If debulking nephrectomy is clinically indicated, MVA should be assessed in the primary site, but otherwise may be assessed at the metastatic site.

A number of clinical studies have reported discordance in tumor shrinkage in primary and metastatic RCC tumors in patients treated with VEGF pathway targeting drugs treated with the primary tumor *in situ*. Abel et al. reported that while tumor shrinkage was seen in primary sites, the degree of shrinkage was smaller than in metastatic sites [44]. Our study showed slightly higher MVA in primary than metastatic sites, but this difference did not reach statistical significance. The smaller radiographic changes in primary tumors than metastatic tumors is more likely due to the mechanism of action of these drugs than differential anti-tumor activity in primary and metastatic sites; the anti-angiogenic effects likely cause necrosis in highly vascular tumors which may not result in large changes in tumor diameter. This hypothesis is supported by the improved progression free survival with drugs such as sorafenib in the setting of a low objective response rate by standard radiographic criteria [10].

Another goal of this study was to assess intra-tumor variability in MVA. We previously reported that in our large cohort of primary tumors studying MVA using the same automated method, intra-tumor variability was negligible, and the MVA obtained from different parts of the nephrectomy specimen was similar (ρ = 0.8) [31]. In the present study we had similar findings; as shown in Figure 2, using the smaller cohort of matched primary and metastatic samples, we validate our previous observations. This suggests that MVA obtained from core biopsies can reflect that of the entire tumor.

Historical concerns about bleeding from biopsies done to diagnose RCC have largely been refuted in recent years. The incidence of bleeding from biopsies from primary renal specimens has been reported to be exceedingly low in recent years, although most series did not evaluate post-biopsy hemorrhage by imaging and did not assess the incidence of bleeding from metastatic tumors [45,46]. While no clear association has been made between tumor vascularity and hemorrhage, our data show that there is no significant difference in vascularity between the primary and metastatic sites, suggesting that tumor vascularity should not be a consideration in deciding anatomic preference for biopsy.

Clear cell RCC represents the most common histologic subtype. Phase III studies of sunitinib, sorafenib, bevacizumab, pazopanib and axitinib excluded non-clear cell histologies [9-12,47]. Subsequent studies, however, showed that these drugs may be beneficial in non-clear cell histologies as well, although the efficacy in papillary RCC appears to be lower than the historically reported response in clear cell RCC [48]. The response rate in the small number of patients in this study with chromophobe RCC was less disappointing. Here we show that vascularity of clear cell RCC is higher than papillary and oncocytoma subtypes, yet the MVA of chromophobe RCC was slightly lower than that of clear cell RCC, but this difference did not reach statistical significance (P = 0.063). The differences in MVA demonstrated with this method may explain the differences in response rate to anti-angiogenic therapies with the different histological subtypes.

## Conclusion

In summary, our data show that MVA within a tumor is fairly uniform, suggesting that MVA measured from a biopsy specimen is may represent that of the entire tumor. Although MVA was slightly higher in primary than metastatic specimens, this difference was not statistically significant, suggesting that if MVA is associated response to VEGF pathway targeting drugs, anti-tumor effects should be seen in both primary and metastatic sites. These studies need to be validated in additional, larger cohorts. While there was a fair correlation between MVA in matched primary and metastatic sites, discordant cases were seen, indicating that future predictive biomarker studies entailing MVA measurements should include specimens from both sites to verify concordance in MVA and further determine the association between MVA and response to anti-angiogenic therapies. Clear cell carcinomas have higher MVA than other histologic subtypes, which may explain the higher response rate to VEGF pathway targeting therapies in clear cell RCC. Further studies of MVA using quantitative measurements such as those used here should be incorporated into clinical trials of anti-angiogenic drugs in RCC.

## Competing interests

RLC is a co-founder, stockholder and consultant for a company called HistoRx that has licensed the technology for automated tissue analysis used in this study.

## Authors’ contributions

SAA and JS performed experiments. HMK and RLC designed experiments. RLC and AA performed pathology review. HMK, SAA and JWC wrote the manuscript. RLC, SAA, JS and HMK performed the statistical analysis. HMK supervised the project. All authors read and approved the final manuscripts.
